# Stability and Change in Diffusion Model Parameters over Two Years

**DOI:** 10.3390/jintelligence9020026

**Published:** 2021-05-12

**Authors:** Mischa von Krause, Stefan T. Radev, Andreas Voss, Martin Quintus, Boris Egloff, Cornelia Wrzus

**Affiliations:** 1 Department of Psychology, Heidelberg University, 69117 Heidelberg, Germany; stefan.radev@psychologie.uni-heidelberg.de (S.T.R.); Andreas.Voss@psychologie.uni-heidelberg.de (A.V.); cornelia.wrzus@psychologie.uni-heidelberg.de (C.W.); 2 Department of Psychology, Mainz University, 55122 Mainz, Germany; mquintus@uni-mainz.de (M.Q.); egloff@uni-mainz.de (B.E.)

**Keywords:** diffusion model, cognitive modelling, individual differences, stability, longitudinal study

## Abstract

In recent years, mathematical models of decision making, such as the diffusion model, have been endorsed in individual differences research. These models can disentangle different components of the decision process, like processing speed, speed–accuracy trade-offs, and duration of non-decisional processes. The diffusion model estimates individual parameters of cognitive process components, thus allowing the study of individual differences. These parameters are often assumed to show trait-like properties, that is, within-person stability across tasks and time. However, the assumption of temporal stability has so far been insufficiently investigated. With this work, we explore stability and change in diffusion model parameters by following over 270 participants across a time period of two years. We analysed four different aspects of stability and change: rank-order stability, mean-level change, individual differences in change, and profile stability. Diffusion model parameters showed strong rank-order stability and mean-level changes in processing speed and speed–accuracy trade-offs that could be attributed to practice effects. At the same time, people differed little in these patterns across time. In addition, profiles of individual diffusion model parameters proved to be stable over time. We discuss implications of these findings for the use of the diffusion model in individual differences research.

## 1. Introduction

Recently, the use of mathematical process models of cognition has seen an upsurge in research on individual differences in cognitive abilities and intelligence (Ratcliff and Childers 2015; Ratcliff et al. 2011; Schmiedek et al. 2007; Schubert and Frischkorn 2020; Voss et al. 2013). It has been proposed that our understanding of intelligence and cognition can profit from such modelling approaches, which disentangle different cognitive processes and components involved in solving cognitive tasks (Frischkorn and Schubert 2018; Schubert and Frischkorn 2020). One crucial aspect when employing mathematical models to estimate cognitive parameters to further our understanding of individual differences is whether these parameters have trait-like properties, that is, whether they measure processes which are stable and consistent across tasks and time.

### 1.1. Brief Introduction of the Diffusion Model

One of the most prominent models of cognition is the diffusion model (Ratcliff 1978). It is a stochastic model for the analysis of response times and accuracy rates in binary decision tasks, for example, a recognition memory task or a lexical decision task. It utilizes the full empirical response time distributions and accuracy rates simultaneously to estimate different parameters, which map onto specific components of the decision process. One of the main advantages of the diffusion model compared to the analysis of mean response times is that it can disentangle these different components. Most notably, speed–accuracy trade-offs can be distinguished, that is, the fact that people sometimes show slower response times because they are more cautious. Among others, the model provides separate estimates of speed of information processing, decision caution (i.e., speed–accuracy trade-off), and the time taken for encoding and motor response processes.

**Figure 1:**
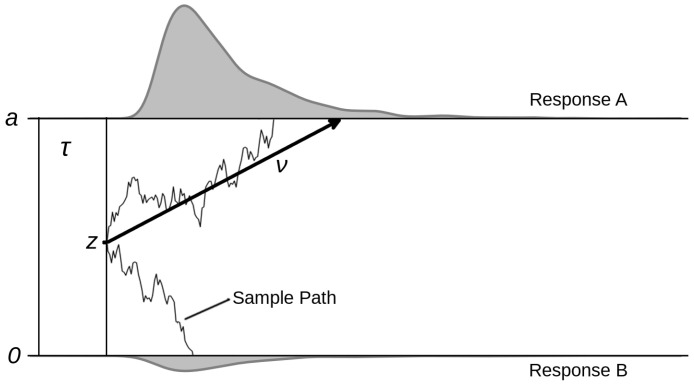
The diffusion model. The accumulation process starts at starting point *z*, moves with average slope ν and terminates when one of the two thresholds (0 or *a*) has been reached. τ denotes the time taken for non-decisional processes, e.g., encoding and motoric response. On the top and the bottom of the figure, the two response time distributions are shown.

Figure 1 depicts the diffusion model and its core parameters. The decision process is modelled as a stochastic sampling of noisy information. The two possible responses are associated with the two decision boundaries named *a* and *0* in the graph. The drift rate (ν) denotes the average speed of information accumulation towards one of the two boundaries. The separation between the two boundaries (*a*) determines how much information is sampled before a decision is taken, that is, when the noisy accumulation process reaches one of the two boundaries. Thus, *a* is a measure of decision conservatism or caution. The starting point, *z*, determines where the accumulation process starts, and maps a possible bias in the decision process in favour of one of the two responses. Finally, the non-decision time (τ) sums the duration of all non-decisional processes. On the top and the bottom of the graph are presented two example response time distributions generated by the model with a fixed parameter configuration. In addition to the parameters described above, the full diffusion model also contains parameters for the across-trial variability in drift rates, starting points and non-decision times, that help explain certain special patterns found in empirical response time distributions, like quick or slow errors (Ratcliff and McKoon 2008; Ratcliff and Rouder 1998).

In the past decades, the diffusion model has been applied in various contexts, for instance, in studies on intelligence (Lerche et al. 2020; Ratcliff et al. 2010; von Krause et al. 2020) or ageing studies (Ratcliff 2008; Ratcliff et al. 2004; Theisen et al. 2020) and has found widespread use especially in the field of cognitive psychology (Ratcliff and McKoon 2008; Voss et al. 2013). One particular question that crosses the boundaries of cognitive research towards the study of individual differences concerns the interpretation of diffusion model parameter estimates. Do they constitute reliable measures of trait-like constructs reflecting meaningful inter- and intra-individual differences between and within persons? A core aspect of traits as defined in the literature is their relative stability across time and measurement methods (Allport 1937; John et al. 2008). While many studies have demonstrated that diffusion model parameters show substantial correlations across different experimental tasks (see, e.g., Lerche et al. 2020; Ratcliff et al. 2010; Schubert et al. 2016), the question of temporal stability has received comparably little attention.

The first published results on the stability of diffusion model parameters were strong test–retest correlations of around r= 0.70 found for all three main diffusion model parameters in a lexical decision task across a time interval of up to one week (Yap et al. 2012). In another study across one week, medium to strong test–retest correlations were observed for the main diffusion model parameters (ν, *a*, τ), with values ranging from r> 0.70 for drift rates and boundary separation and r> 0.40 for non-decision time (Lerche and Voss 2017). Schubert et al. (2016) conducted a systematic study of the trait properties of diffusion model parameters over eight months, utilizing two different response time tasks and analysing them via latent state-trait structural equation models. The results showed stability across both tasks and time for all three main diffusion model parameters, with speed of information processing (drift rate) showing the highest stability and consistency: the latent trait factor generalizing over both time points and both tasks on average accounted for 44% of the manifest variance in drift rate. Task-specific across time correlations ranged from r= 0.44 to r= 0.71 for drift rates, from r= 0.20 to r= 0.60 for boundary separations and from r= 0.26 to r= 0.63 for non-decision times (Schubert et al. 2016). These results suggest that some diffusion model parameters show considerable stability at least over the range of one week to eight months and might therefore in this regard be characterized as trait-like. However, the findings warrant further research, because rank-order correlations across time are only one aspect of stability.

### 1.2. Different Forms of Stability and Change in Individual Differences

While the notion of temporal stability remains a core feature of classical as well as contemporary definitions of personality traits (Allport 1937; John et al. 2008), the idea that traits are essentially fixed at a certain point in life and remain stable thereafter has come under more and more scrutiny in the past two decades (Wagner et al. 2020). Thus, it is now commonplace to study different forms of stability and change in personality traits to better understand their development over time.

One approach to studying stability and change that has found considerable echo in the literature was described by Roberts et al. (2008). Mainly referring to the Big Five, they proposed to study four aspects of stability and change. First, rank-order stability (i.e., in most cases, test–retest correlations) refers to the stability of people’s relative positions to others on the trait continuum. Second, mean-level change is the development of average (i.e., across person) levels in a certain trait over time. For example, people tend to become more agreeable and conscientious during young adulthood (Roberts et al. 2006). Third, individual differences in change refer to the individual deviations in developmental patterns from the mean-level change in the sample. Finally, profile stability refers to the stability of the relative patterns of traits within a person across time: a person might stay more extraverted than she is agreeable, although both traits show changes in their absolute values. While the different forms of stability and change suggested by Roberts et al. (2008) have (to different degrees) been extensively studied for Big Five traits, the literature on diffusion model parameters has so far focused solely on rank-order stability over two time points.

In the present paper, we expand the scope of previous longitudinal studies of the diffusion model, and report findings on relative stability, mean-level change, individual differences in change and profile stability in the main diffusion model parameters across four time points over two years.

We focus on a specific decision task that the diffusion model has repeatedly been applied to: the Implicit Association Test (IAT; Greenwald and Farnham 2000; Greenwald et al. 1998, 2003; Klauer et al. 2007). In the IAT, participants make binary decisions, typically classifying presented stimuli into one of two categories. In general, there are two different classification tasks (e.g., old vs. young, quick vs. slow) that are combined in some blocks of the experiment to form so-called congruent (e.g., old/slow) and incongruent (e.g., old/quick) combinations. The difference in mean response times between the congruent and incongruent block is then interpreted as a measure of the implicit association between the corresponding constructs (e.g., age and speed). The IAT has also been employed as a measure of implicit personality (Nosek et al. 2007). In this case, the classification categories are, for instance, “extraverted” vs. “introverted” on the one hand, and “me” vs. “other” on the other hand. The difference in response times between the blocks combining “me” and “extraverted” versus those combining “me” and “introverted” is then interpreted as a measure of implicit extraversion (Back et al. 2009).

When applying the diffusion model to the IAT, differences in performance can be decomposed into differences in speed of information processing (ν), differences in decision caution (*a*), and differences in non-decision time (τ). Previous studies have shown that the IAT effect can mostly be attributed to differences in ν that are strongly linked to the *D* scores usually employed to estimate the IAT effect (Klauer et al. 2007). At the same time, there were also differences in *a* and τ for the congruent and incongruent blocks (Klauer et al. 2007; van Ravenzwaaij et al. 2011). Thus, the IAT could be an interesting example to study the stability and change in diffusion model parameters. It can easily be analysed with the diffusion model and such analyses improve the understanding of the underlying processes when working on the task. The focus of this paper is, however, not on the task-specific aspects and interpretation of the IAT, but on the longitudinal analysis of diffusion model parameter estimates as cognitive process parameters involved in the IAT. Namely, in our analyses we set aside the effects of the conditions (though we do include them in our model), and study the across-task and across-block estimates of the parameters. In this way, we account for the specific effects of each IAT condition and task, while keeping the results focused on the overall cognitive processes, and the number of analyses circumscribed.

### 1.3. The Present Study

In this paper, we analyse the stability of the diffusion model’s measures for speed of information processing (drift rate), decision caution (boundary separation) and non-decision time using data from an implicit personality IAT across four time points over a period of two years. We employ state-of-the-art hierarchical Bayesian diffusion modelling in order to represent the hierarchical structure of the data, maximize information utilization and obtain principled uncertainty estimates. To our knowledge, this is the first study to assess the development of diffusion model parameters over more than two time points and over such an extended time period. We conducted analyses addressing four forms of stability and change: rank-order stability, absolute mean-level change, individual differences in change and profile stability, all with respect to drift rate (ν), boundary separation (*a*), and non-decision time (τ), to receive a comprehensive picture of stability and change in the cognitive parameters derived from the diffusion model.

## 2. Methods

### 2.1. Participants

The data used in this paper were collected in a large-scale longitudinal study that focused on temporal aspects of personality. This study included a wide range of measures of explicit and implicit personality traits, personality states and cognitive abilities. Several papers drawing on these data have already been published (Lücke et al. 2020; Quintus et al. 2017, 2020). These studies emphasized different aspects of personality processes and personality development. However, none of these papers focused on cognitive parameters or used the diffusion model in any of the analyses.

We recruited 382 participants via local newspapers, flyers in public places (cafés, drug stores, vocational schools), Facebook-groups, mailing lists and from introductory non-psychology courses for regular and senior students at the university of Mainz, Germany (see Quintus et al. 2017). Participants received a compensation of up to 117 € for completing the full study protocol, which also included up to 50 daily assessments (see Quintus et al. 2020).

The initial sample at the first time point (T1) comprised 382 participants (73% women, all with a similar educational background, the German Abitur). Of these, 255 were young adults ($M_{age}=21.57$, $SD_{age}=2.20$, $Min_{age}=17$, $Max_{age}=32$) and 127 were older adults ($M_{age}=67.76$, $SD_{age}=5.31$, $Min_{age}=52$, $Max_{age}=84$). The sample size was based on power analyses independent of the analyses reported in this paper. After six months (T2), 358 people from the original sample took part in the second time point. Both at T3 (one year after T1) and at T4 (two years after T1), 327 people participated. The sample consisted of five different subgroups: young people in their first year at university (Group 1, n=113 at T1), young people in their second year at university (Group 2, n=109), young non-students (Group 3, n=26), older first-year students (Group 4, n=63), and older non-students (Group 5, n=58).

### 2.2. Procedure and Material

Laboratory data were collected in small age-homogeneous group sessions on a PC in a university setting. All participants provided informed consent. The first session (at time 1) took up to two hours, with initial questionnaires already provided to participants via mail. Sessions three and four that were conducted online and at home took approximately one hour. Participants worked on the IATs halfway through the sessions, with questionnaires as filler tasks given after two IATs (i.e., for two traits). In addition, participants were prompted to take breaks after completing each IAT. As was already mentioned, the study included a wide range of measures, most of which focused on personality traits and states. An overview of the instruments employed is available at https://osf.io/k9wsv/ (accessed 11 May 2021). In the following, we describe the Implicit Association Tests of the Big Five personality traits.

The Big Five IATs (Schmukle et al. 2008) include five blocks of word classification tasks, with 20 trials in all training blocks and 60 trials in both the congruent and the incongruent test blocks, as is standard practice in IATs (Greenwald et al. 1998, 2003). Since we disregarded the practice trials in our analyses, this led to a total trial number of 600 per participant and time point (60 × 2 [congruent/incongruent] × 5 [Big Five traits]). For all Big Five traits, the same target categories (i.e., “me” and “others”) were used with a set of five different stimuli each (e.g., “I”, “they”). Attribute category labels were dependent on the specific Big Five traits (e.g., “conscientiousness” vs. “carelessness”) and also included five different stimuli for each of the traits (e.g., “helpful” for agreeableness or “reliable” for conscientiousness). In all blocks, stimuli were always presented in random order and then shuffled before the next presentation. In the test blocks, we alternated target and attribute stimuli. One notable characteristic of the IAT data was the way error response times were recorded. The stimuli remained on screen until the correct response was given. In case of an error response, the trial did not terminate until the participant had corrected their response. The latter was recorded along with an indicator variable for the erroneous response. This coding is typical for IAT analyses but presents a particular challenge for diffusion model analysis. This is important for the modelling approach we used, since we tried to account for the differences in processes involved in creating the correct and error response times.

### 2.3. Data Analysis

We used the programming language R (Version 4.0.3; R Core Team 2020) and the R-packages *BayesFactor* (Version 0.9.12.4.2; Morey and Rouder 2018), *blavaan* (Version 0.3.12; Merkle and Rosseel 2018), *correlation* (Version 0.5.0; Makowski et al. 2020), *papaja* (Version 0.1.0.9997; Aust and Barth 2018) and *tidyverse* (Version 1.3.0; Wickham et al. 2019) for all statistical analyses. For all Bayesian analyses, the prior distributions used are available in the Appendix A. For the diffusion model parameters, we chose the default priors provided by the Python package *HDDM* (Wiecki et al. 2013), which are based on the recommendations by Matzke and Wagenmakers (2009).

#### 2.3.1. Estimation of the Diffusion Model Parameters

We used the hierarchical Bayesian method provided in *HDDM* (Wiecki et al. 2013) to estimate the diffusion model parameters. Prior to fitting the models, we removed trials that had not been recorded for technical reasons and also trials with latency below 300 ms or above 3000 ms, as these could be expected to qualitatively differ from the other trials regarding the processes involved in producing the answers. Separately for each time point, we also excluded all data from participants with low accuracy (across all five Big Five IATs). Low accuracy was defined as an accuracy rate lower than three interquartile ranges from the first quartile of accuracy rates across participants per time point (Tukey 1977). Taken together, these pre-processing steps lead to the exclusion of 2.91% of the total number of trials. Finally, we excluded one warm-up trial per block per participant.

We fitted the same model separately for each time point. Using the Marcov chain Monte Carlo method implemented in *HDDM*, we obtained four chains with 6000 samples each from the posterior distribution per model. We discarded the first 1000 samples of each chain as a burn-in period. For all diffusion model parameters, we obtained posterior distributions both at group-level and at the person-level. We choose a parsimonious modelling approach, including only the core diffusion model parameters: drift rate, boundary separation, and non-decision time. The estimates of between-trial variability of the parameters are often unreliable and estimating them can actually have detrimental effects on the reliability of the main parameter estimates (Lerche and Voss 2016). Thus, we fixed these parameters to zero, as they were also of no theoretical interest for our analyses. We also fixed the starting point to 0.5, as the decision boundaries were associated with correct and error responses and thus no implicit bias towards one of the alternatives could be expected.

**Figure 2:**
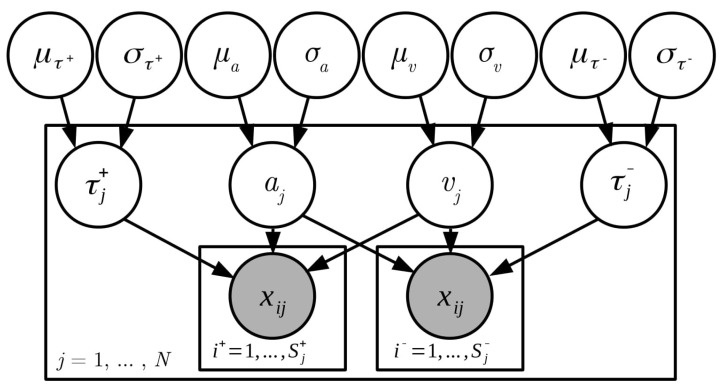
The hierarchical Bayesian model used for estimation of the diffusion model parameters. The inner plates relate to the trial level, the outer plate to the person level. On the outside are the group-level parameters. ν= drift date, a= boundary separation, $\tau^{+/-}=$ non-decision time for correct and error responses, N= number of participants at a certain time point, $S^{+/-}$ = number of correct/error trials per person. $x_{ij}$ denotes a single trial. The figure does not show the effects on drift rate and boundary separation estimated at the group-level and person-level for the different experimental conditions and traits.

To model the different experimental conditions (i.e., the five different Big Five traits, both in the congruent and the incongruent block), we used effect coding to estimate an intercept and effects per condition for both boundary separation and drift rates. Further, different non-decision times were estimated for correct and error responses. This was necessary, as the latency for the initial (erroneous) response was not recorded, but only the time of the corrected response. In our model, the time to correct the response is included in the error non-decision time. We assume that the time it takes to give the additional corrected response can be thought of as an additive constant that is part of the non-decisional processes contributing to error response times. Figure 2 depicts our model formulation.

To ensure convergence of the Markov chains to the target posterior, we used several steps to inspect the group-level and individual parameters of drift rates, boundary separations and non-decision times (Kruschke 2015). First, we visually inspected each chain via caterpillar plots. Second, we checked the $\hat{R}$ statistics and excluded estimates with a $\hat{R}$ value larger than 1.01 (Vehtari et al. 2020). Third, we computed the bulk effective sample sizes and excluded estimates with fewer than 400 effective samples (i.e., 100 per chain). To obtain full sets of the main diffusion model parameters for each participant at each time point, we excluded the individual parameter estimates of all of *a*, ν and the two τs if signs of non-convergence were evident for any of these four parameters in a person (at a certain time point). Taken together, all preprocessing steps led to the exclusion of 7.44% of the total individual parameter vectors. The corresponding statistics and plots can be found in the supplementary material.

**Figure 3:**
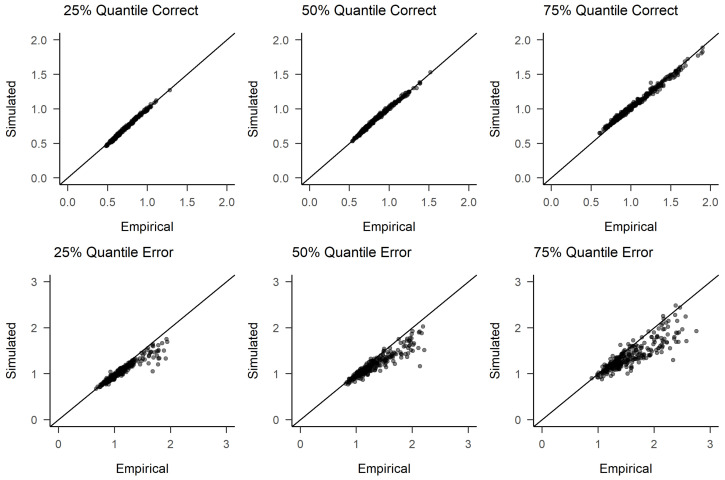
Posterior predictive check of RTs for T1. Error quantiles are based on far less data, with the median accuracy rate being 96 percent. Participants with 10 or less errors are omitted from the error response time plots. See Appendix A for posterior predictive checks for the other time points.

To further assess model fit (generative performance), we conducted posterior predictive checks. For each time point, we randomly selected 500 samples from the joint posterior distribution of parameters and used each of these to generate person-specific simulated response times and response choices. As in the empirical data, 600 trials existed for each person at each time point (unless outlier trials had been removed as described above), we also obtained 600 trials per person for each of the 500 samples from the posterior distribution of diffusion model parameters (i.e., 60 for each of the trait/condition combinations with their specific effects). We then computed RT quartiles and error rates for each person and time point from both the empirical and simulated data. Figure 3 shows the resulting scatter plot of RTs for T1, the remaining plots can be found in the Appendix A. As can be seen, the patterns found in the observed data closely match those found in the simulated data, indicating an adequate model fit. This seems especially noteworthy given the fact that we used a parsimonious model, ignoring possible across-trial variabilities in drift rates or non-decision times. Our theoretically grounded model thus seems to achieve a good balance between parsimony and goodness of fit.

Following model evaluation, we extracted, for each time point, each person’s individual posterior medians for the three diffusion model parameters. We used the intercept parameter estimates irrespective of condition and trait for *a* and ν, and the non-decision times of correct responses. We did not further analyse error non-decision times because estimates were based on a low number of trials. We then utilized these posterior medians as summaries of the full posteriors in most of the further analyses. While it is true that such a two-step procedure makes no use of uncertainty estimates provided by Bayesian sampling procedures, it must be noted that our models already contained several thousands of parameters to be estimated for each time point and were thus very complex to estimate and converge.

To account for possible drop-out effects also due to non-converged chains only at later time points, we conducted Bayesian *t*-tests addressing whether the persons who had missing values at at least one of the later time points differed from the rest of the sample in any of the three diffusion model parameters. People with missing values had higher drift rates (BF=5.86), higher boundary separation (BF=3.24) and higher correct non-decision times (BF=195.03). To account for this fact, we repeated all our analyses including the non-converged chains. No differences in the pattern of results emerged, notably also not for the pattern of mean-level changes across time. In addition, when not excluding the non-converged chains, there were no more differences in means of diffusion model parameter for people dropping out (all BFs<1).

#### 2.3.2. Statistical Analyses of Stability and Change

To test the **rank-order stability** of the diffusion model parameters, we obtained Bayesian correlation estimates (between individual posterior medians). Hypothesis testing was performed with Bayes factors (instead of *p* values) using the *R* packages *correlation* (Makowski et al. 2020) and *BayesFactor* (Morey and Rouder 2018). As the sample contained different sub-groups of participants (old/young, student/non-student, see above), we conducted separate analyses for each of the sub-groups to study whether the overall rank-order stability between participants might be due to the stability of differences between sub-groups. To analyse **mean-level change**, we compared the full posterior distributions of the group-level parameter estimates (i.e., across participants) across time points.

**Figure 4:**
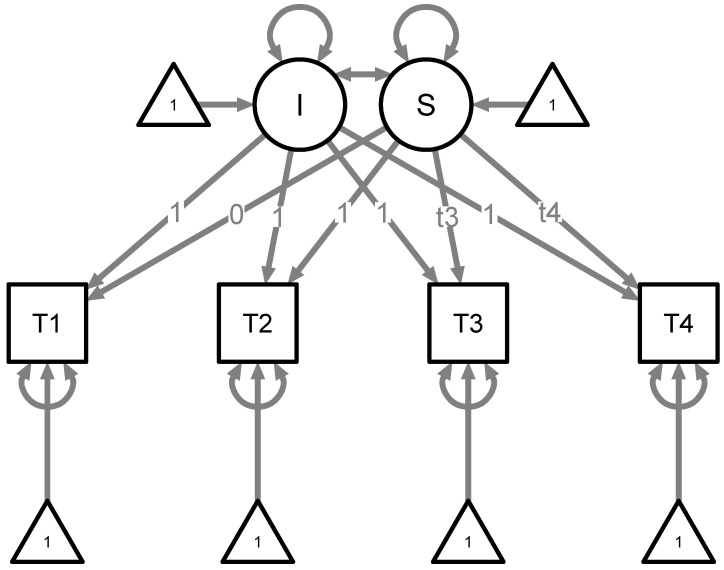
Growth curve model used for all three diffusion model parameters. T1 to T4 refer to the individual posterior medians of the respective diffusion model parameter at a certain time point. *I* = Intercept, *S* = Slope. The slope loadings t3 and t4 are treated as free parameters and thus estimated.

To study possible **individual differences** in stability and change in diffusion model parameters, we then estimated Bayesian growth curve models using the *blavaan* package (Merkle and Rosseel 2018), separately for each parameter (ν, *a* and τ). The individual posterior medians at each time point served as observed variables in the model. We fixed all (unstandardized) loadings on the intercept factor to 1. For the slope factor (which reflects growth or change over time), we fixed the loading to 0 for T1 and to 1 for T2. We freely estimated the factor loadings for T3 and T4, as we did not have any hypotheses on the nature of change. Figure 4 shows a graphical representation of our growth curve models. For each of the models, we used three MCMC chains and obtained 10000 samples, discarding the first 5000 samples as burn-in (Merkle and Rosseel 2018). To check the fit of the Bayesian growth curve models, we inspected the *bCFI* and *bGammaHat* metrics as advised by Garnier-Villarreal and Jorgensen (2020).

Finally, we calculated *q* correlations of individual posterior medians to study **profile stability** (Burt 1937). In the *q* correlation framework, variables (i.e., ν, *a*, and τ) serve as cases which vary in relative strength and time points constitute the columns in separate datasets for each participant. In this way, it is possible to calculate the stability of the relative strength of the values (i.e., ν, *a*, and τ), compared to one another. To this end, we first *z*-standardized the individual posterior medians, separately for each parameter, to make their relative strength comparable. We then calculated (frequentist) *q* correlations via the *multicon* package, separately for each participant, and created descriptive statistics and plots of correlations across participants. In order to reflect the exploratory nature of these calculations, we do not conduct inferential analyses of *q* correlations, but purely report the descriptive results.

## 3. Results

All data and analysis scripts can be found on the paper’s OSF page (https://osf.io/cnr2a/, accessed on 11 May 2021). We report results on the rank-order stability, mean-level change and individual differences in change for each of the three main diffusion model model parameters (ν, *a*, τ). For all these analyses, we used Bayesian methods to obtain our results. We also conducted all analyses using a frequentist, *p*-value based approach. This did not alter the interpretation of our findings. Finally, we report findings on the profile stability of the three parameters across time.

Table 1 shows the descriptive statistics of the individual posterior medians for the three diffusion model parameters for each of the four time points across the entire sample. Table A2, Table A3, Table A4, Table A5, Table A6 in the Appendix A contain the corresponding information, split up for each of the five sub-groups.

### 3.1. Rank-Order Stability

Table 2 shows the rank-order stability estimates of the diffusion model parameters for the entire sample. We report Bayesian correlation estimates, using a uniform prior for the correlation (see Table A1) and individual posterior medians as variables. Rank-order stability was high for drift rates (ν; all rs>=0.64) across the entire time span, with correlations getting slightly smaller for larger time periods (e.g., r=0.79 from T1 to T2, but only r=0.64 from T1 to T4). We found the same pattern for boundary separation (*a*): Rank-order stability was high (all rs>=0.83), with correlations getting slightly smaller across larger time periods (e.g., r=0.90 from T2 to T3, but only r=0.83 from T1 to T3). For non-decision times (τ), stability was again high (all rs>=0.80) across the entire time span, with correlations once more getting smaller for larger time periods (e.g., r=0.90 from T2 to T3, but only r=0.80 from T1 to T4). All correlations showed Bayes factors >999 when compared to a null-model.

Table A7, Table A8, Table A9 show the estimates of rank-order stability separately for the three diffusion model parameters and split up across the five sub-groups studied. Generally, the interpretation of the pattern of results did not differ across groups, although within-group correlations often were slightly smaller than correlations for the total sample. Especially due to the smaller samples sizes, Bayes factor were also sometimes lower, for example, as low as BF=3.07 for the correlation of drift rates at T2 to the ones at T4 in Group 3 (n=19, r=0.46).

### 3.2. Mean Level Change and Individual Differences in Change

**Figure 5:**
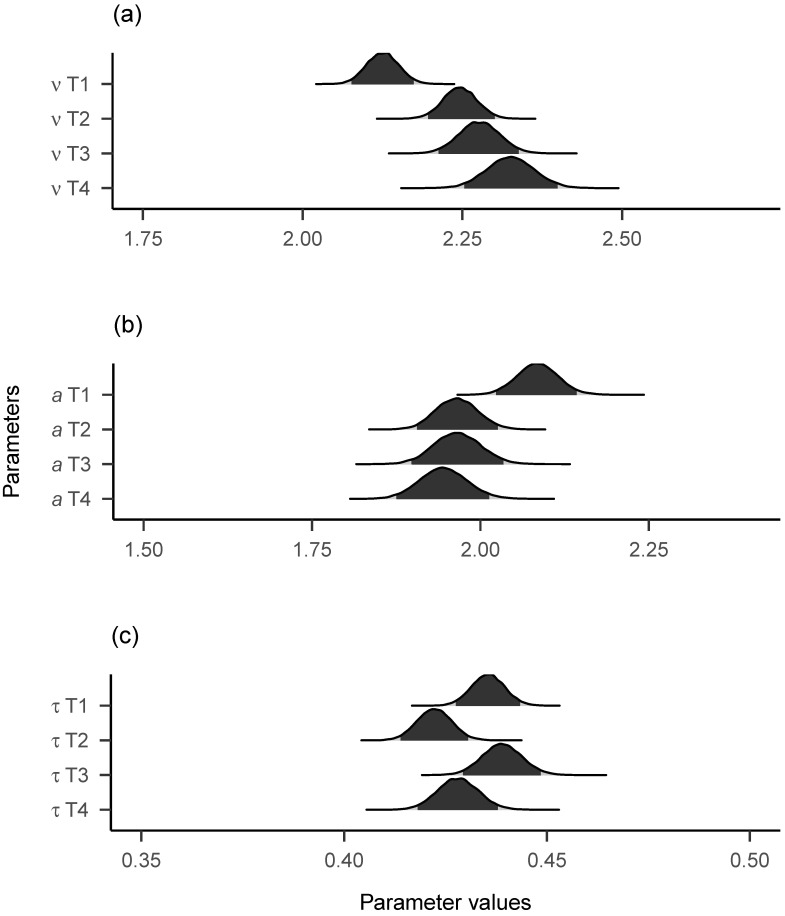
Group-level posterior plots of diffusion model parameters across time. (**a**) = Drift Rates. (**b**) = Boundary Separations. (**c**) = Non-Decision Times. The 95% highest density intervals are shown. T2 = T1 + 6 months. T3 = T1 + 12 months. T4 = T1 + 24 months.

Figure 5 shows the group-level posterior distributions (i.e., across participants) for the three diffusion model parameters across the four time points. As can be seen, drift rates seem to rise after T1 (with the corresponding 95% highest density interval (HDI) showing no overlap with those of the other time points) and to a lesser degree also after T2 and T3. The pattern reverses for the boundary separation parameter, with a decline from T1 to the later time points. For non-decision times, no clear pattern of mean level change is evident. It should be noted that the group-level posterior distributions are not equivalent to the means of individual parameter posterior medians, due to the hierarchical modelling approach and due to the exclusion of individual parameter estimates with non-converged traces. However, the general pattern of results was the same for both group-level posteriors and means of individual posterior medians.

Table 3 shows the parameter estimates and fit indices for the Bayesian growth curve model of drift rates. The latent intercept and latent slope exhibited only a very weak estimated correlation, indicating that drift rates at T1 did not relate to the developmental patterns of drift rates. As the 95% CI of the covariance between intercept and slope included zero, we fixed this parameter to zero to help model convergence. All estimated parameters had effective sample sizes >5000 and $\hat{R}$ values below 1.01, indicating that the chains had converged. Furthermore, model fit was good according to the mean Bayesian GammaHat estimate >0.99 and the mean Bayesian CFI estimate >0.99.

Latent slope loadings at T3 and T4 were estimated as 1.142 and 1.297. Both the mean level (intercept) of the latent intercept parameter and of the latent slope parameter were estimated as positive and their 95% credibility intervals (CIs) did not include zero. This indicates that drift rates were generally positive at T1 (as would be expected) and tended to increase over time. The latent intercept showed considerable variance, indicating that people differed in their speed of information accumulation at T1. The latent slope parameter also indicated variance, meaning that people differed in their developmental patterns of drift rates across time—the 95% CI did not include zero.

Table 4 shows the parameter estimates and fit indices for the Bayesian growth curve model of boundary separations. The latent intercept and and latent slope exhibited only a very weak estimated correlation, indicating that boundary separation at T1 did not relate to the developmental patterns of boundary separation. As the 95% CI of the covariance between intercept and slope included zero, we fixed this parameter to zero to help model convergence. As the variance of the slope factor was also estimated to be zero and the model showed divergent transitions when estimating it, we also fixed this parameter. All estimated parameters had effective sample sizes >5000 and $\hat{R}$ values below 1.01, indicating that the chains had converged. Model fit was good, with the mean Bayesian GammaHat estimate >0.99 and the mean Bayesian CFI estimate >0.99.

Latent slope loadings at T3 and T4 were estimated as 1.233 and 1.334. The mean level (intercept) of the latent intercept parameter was estimated as positive, while the mean level (intercept) of the latent slope parameter was estimated as negative. Both their 95% CIs did not include zero. This indicates that boundary separations were generally positive at T1 (as would be expected) and tended to decrease over time. The latent intercept showed considerable variance, indicating that people differed in their decision criteria at T1. As was already mentioned, the latent slope parameter was estimated and then fixed to be zero.

Table 5 shows the parameter estimates and fit indices for the Bayesian growth curve model of non-decision times. Latent intercept and latent slope showed a very low estimated correlation, indicating that non-decision time at T1 did not relate to the developmental patterns of non-decision times. As the 95% CI of the covariance between intercept and slope included zero, we fixed this parameter to zero to help model convergence. As the variance of the slope factor was also estimated to be zero and the model showed divergent transitions when estimating it, we also fixed this parameter.

All estimated parameters had effective sample sizes >5000 and $\hat{R}$ values below 1.01, indicating that the chains had converged. Model fit was good, with the mean Bayesian GammaHat estimate >0.97 and the mean Bayesian CFI estimate >0.98.

Latent slope loadings showed an unclear pattern, with loadings at T3 and T4 estimated as −0.358 and 0.509. The mean level (intercept) of the latent intercept parameter was estimated as positive, while the mean level (intercept) of the latent slope parameter was estimated as negative. Both their 95% CIs did not include zero. This indicates that non-decision times were generally positive at T1 (as would be expected). Given the unclear pattern of loadings on the slope factor, no clear interpretation of the negative intercept of the latent slope factor emerged. The latent intercept showed considerable variance, indicating that people differed in their non-decision time at T1. As was already mentioned, the latent slope parameter was estimated and then fixed to be zero.

In summary, we found notable individual differences in growth curve model intercepts for drift rates, boundary separations, and non-decision times. Regarding growth curve model slopes (i.e., rates of change), we only found individual differences for drift rates, but not for boundary separations or non-decision times.

### 3.3. Profile Stability

**Figure 6:**
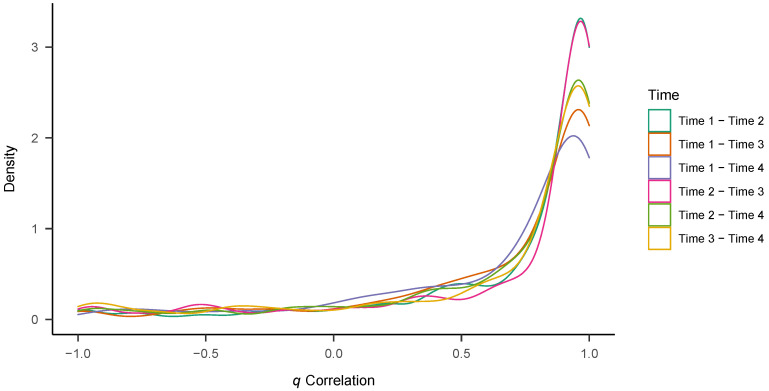
Density plots of *q* correlations.

We estimated *q* correlations of the *z*-standardized individual posterior medians for the three diffusion model parameters across all possible combinations of time points (T1 with T2/T3/T4, T2 with T3/T4, T3 with T4). Table 6 shows the means, standard deviations, and medians across participants. Profile stability was generally high, with all median *q* correlations >0.85. However, there was also considerable variance in correlations across participants (all *SD*s >0.42), with lower mean correlations than median correlations. Figure 6 shows density plots of the individual *q* correlations for all six periods. As can be seen, a large part of the densities lies close to 0.95, but there are also much lower coefficients of stability and also participants showing negative *q* correlations.

## 4. Discussion

In this article, we studied stability and change of cognitive processes as measured by the three main diffusion model parameters-processing speed (i.e., drift rates), decision caution (i.e., boundary separations), and speed of encoding and motor response (i.e., non-decision times), using four different indices of stability and development. To our knowledge, this is the first study to analyse diffusion model parameters (i) over such a long time period, (ii) across more than two time points, and (iii) in such a large, heterogeneous sample (n=359 at Time 1). Moreover, our main statistical analyses relied on modern Bayesian estimation methods which offer multiple advantages compared to traditional methods. Overall, our analyses aimed to investigate whether the cognitive constructs encoded by diffusion model parameters exhibit a measurable trait-like nature. In the following, we briefly summarize the gist of our results.

Regarding rank-order stability, we found robust temporal stability of the main diffusion model parameters. Generally speaking, temporal correlations were high for all three parameters. This held true even when the entire period of the study (i.e., two years) was considered. The correlations we found were in many cases markedly higher than those previously reported in the literature (Lerche and Voss 2017; Schubert et al. 2016; Yap et al. 2012). Especially for non-decision times, previous studies had sometimes found rank-order stability to be low (r<0.50 across one week in Lerche and Voss 2017). In contrast, our results indicate that non-decision times show even higher correlations across long time periods (rs>0.80) than drift rates. This finding is worth discussing, since drift rates have so far been considered as the most “trait-like” parameters of the diffusion model (Schubert et al. 2016).

The latter difference might be attributable to several features of our study. First, in contrast to previous studies, we employed Bayesian hierarchical diffusion model estimation methods that in the past have been found to provide more robust results in correlational studies (Ratcliff and Childers 2015; Wiecki et al. 2013). Bayesian methods incorporate prior knowledge on probable parameter values. Hierarchical Bayesian methods make use of shrinkage of the individual parameter estimates towards the group-level posteriors, balancing out extreme individual parameter estimates that might reflect noise in the data (Kruschke 2015).

Second, we used a comparatively large number of response times for each participant at each time point (600 trials), which necessarily leads to more precise estimates. Finally, our sample included a large number of participants and exhibited a greater heterogeneity, especially in relation to age. The variance of parameter estimates might account for the higher correlations. However, it must be noted that correlations remained strong-though sometimes notably lower or even within sub-groups as small as around 20 participants (see Appendix A). Thus, the present results cannot be attributed solely to sample size and sample heterogeneity. In the end, our estimates of (correct) non-decision times might be more reliable than the ones reported in previous studies, while boundary separation values might have already been estimated very reliably there. Conversely, drift rates might not show greater stability than in previous studies because of the specific content of the task: differences in drift rates also reflect differences in implicit personality, as their developmental patterns were the original focus of the study.

When looking at the raw data, rank-order stabilities of mean accuracies and median correct and error response times are also quite high (*r* posterior means between 0.77 and 0.96, see Table A10), which speaks in favour of the assumption that our high number of trials per person enables us to obtain reliable parameter estimates. At the same time, it is interesting to note that the stabilities of the diffusion model parameters might jointly contribute to the very high across-time stability of the raw data summary statistics.

Regarding mean-level stability and change, we found evidence for systematic changes in both drift rates and boundary separations. Group-level drift rates increased from the first time point to the second time point six months later. The pattern of increase continued throughout the next two time points, but the posterior distributions showed much overlap there. The increase in drift rates might be interpreted as a practice effect. People tended to process the information needed to solve the IAT tasks more efficiently after they had completed the first time point. Conversely, group-level boundary separations decreased from the first to the second time point and to a lesser degree (once more marked by overlap in the posteriors) thereafter. That is, people tended to apply more liberal decision criteria and gathered less information until they made their decisions in the second to fourth time points. We suppose that participants reduce their decision caution at later time points mainly in response to the increased drift rate: that is, participants notice that they may lower their response criteria without deteriorating accuracy. Additionally, a decrease in accuracy motivation over time might also contribute to the reduction of decision caution.

In the literature on the diffusion model, practice effects in the form of increasing drift rates and decreasing boundary separations (but sometimes also non-decision times and shifting starting points) have repeatedly been reported (Dutilh et al. 2009, 2011; Evans and Brown 2017; Lerche and Voss 2017; Petrov et al. 2011). However, none of these previous studies focused on training effects across such long time periods as in our study, but investigated primarily within-session training effects. It is interesting to note that training effects seem to be stable over months. Evans and Brown (2017) found that people often first adopt non-optimal decision criteria when working on a new task, that is, they are overly cautious and try to avoid mistakes, as is mirrored in high boundary separation in the diffusion model. Having practiced the task many times, people then adapt more lenient decision criteria that are closer to the optimum. Thus, a possible interpretation of our results states that people tend to keep the more lenient decision criterion when returning to the task months or even a year later.

Finally, we did not find systematic changes in non-decision times. Group-level posterior distributions remained roughly the same across the two year time period studied. This is in contrast to the results found in earlier studies on training effects that sometimes found decreasing non-decision times (Dutilh et al. 2009, 2011). Task-specific aspects of the IAT might be responsible for our findings. For instance, Dutilh et al. (2011) found that the effects on non-decision times were partly task-specific as well as item-specific.

Regarding inter-individual differences in intra-individual change, our growth curve models indicate that inter-individual differences are mainly based on across-time intercepts: We found substantial variance in the latent intercepts of all three diffusion model parameters. For boundary separation and non-decision times, people varied in their intercepts (which contribute equally to all time points) but not in their slope parameters, which reflect the rate of change across time. The slope parameter for boundary separation showed a negative trend; this means that the decrease in boundary separation, that is, the use of more liberal decision criteria, is close to universal in our data. As the estimated slope factor loadings in the non-decision time model mirror the unclear and mostly stable group-level trends found for this parameter, the slope factor is hard to interpret. In any case, its variance was estimated to be zero. The slope factor in the drift rate growth curve model was the only slope factor to show substantial inter-individual differences.

Thus, people seem to differ in the ways they profit from training effects in terms of task-related information processing. In post-hoc analyses, we regressed the slope factor on age and found a clear and strong positive correlation. This means that older people tended to increase their drift rates more than their younger counterparts. As older adults did not show lower mean level drift rates (Ratcliff et al. 2004; Schubert et al. 2020; von Krause et al. 2020), this implies that they generally profited more from practice. Of course, these post-hoc analyses must be interpreted cautiously and warrant further developmental research. To sum up, people tended to show great inter-individual differences in their overall levels of drift rates, boundary separations and non-decisions time, but differed little in their developmental patterns, with the exception of drift rates. It would be interesting to follow up on these results in a longitudinal study with a stronger focus on training effects, as these were only of periphery interest here.

Regarding profile stability, the estimated *q* correlations were strongly positive across time in the majority of cases, but not in all. We also found a considerable across-participant variance in correlations, with some people showing *q* values close to zero or even negative. Correlations tended to get lower across larger periods of time. The profiles comprising the relative strengths of drift rate, boundary separation and non-decision might be seen a configuration of process components that together lead to certain empirical response time distributions and accuracy rates. For example, the same accuracy data could be the results of high drift rates and low boundary separation, and vice-versa. In a similar way, some people might show low boundary separation in combination with high drift rates, others in combination with low drift rates. It seems that, for most participants in the study, this parameter configuration remained very much the same across time.

All in all, we found that the three main diffusion model parameters are broadly consistent across time, thus fulfilling a central prerequisite of being identified as traits. This is particularly interesting as the diffusion model can be applied to a large range of binary decision tasks (not just from the cognitive domain). Our results reveal positive change in drift rates and negative change in boundary separation, but little individual differences in change, with the exception of drift rates. Profiles of the three parameters were also quite stable.

### 4.1. Limitations

While our study has a number of unique features, for instance, the distinction between the four forms of stability and change, the four time points over a period of two years, and the relatively large sample size, it also has some limitations. First, the variety of tasks was rather restricted. While we used five different IATs and combined them to obtain task-general parameter estimates, we did not use any other tasks. It is known that diffusion model parameters obtained in different tasks sometimes show only weak correlations among each another (Lerche et al. 2020; Ratcliff et al. 2010; Schubert et al. 2016). Thus, some of the results presented here might be specific to the tasks studied.

Second, it must be noted that the posterior predictive checks did not perfectly recover the error response time distributions. Several different factors might contribute to this. First of all, due to the small number of errors, the empirical quantiles are numerically unstable and thus may not be a good representation of the actual (latent) distribution. Additionally, due to the low number of error responses per person, the group-level parameter of error non-decision times greatly influenced the estimates of individual error non-decision times (because of hierarchical shrinkage). This means that individual deviations in error non-decision times might sometimes have been underestimated. In turn, this might have led to a situation where our approach of modelling error response times with a separate non-decision time parameter was less successful among the very slow errors. Nevertheless, as the focus of this paper is on the psychometric properties and developmental patterns of diffusion model parameters, the relative misfit of this small proportion of trials is of secondary importance.

Finally, there are alternative plausible ways to analyse the present data within a purely Bayesian framework. Intuitively, the most straightforward way to approach the question would have been to formulate and fit a full hierarchical model with time included as an additional level. However, despite being intuitive from a Bayesian lens, such an approach involves an enormous computational cost due to the large number of posteriors that need to be estimated simultaneously. In fact, estimating the full hierarchical model turned out to be practically infeasible using the available computational software. Thus, our two-step approach using posterior medians as summary statistics might underestimate the epistemic uncertainty around parameter estimates. However, we deem our approach a reasonable trade-off, since it incorporates more information than frequentist approaches used in most of the diffusion model literature. Further, it also utilizes hierarchical shrinkage within each time point, thereby rendering point and uncertainty estimates more robust than a non-hierarchical approach.

### 4.2. Conclusions

We examined four different forms of stability and change in the three main diffusion model parameters: drift rate, boundary separation, and non-decision time. Our main aim was to study whether and in which way the assumption of temporal stability that is inherent in the interpretation of model-parameters-as-traits holds. Across a time period of up to two years, all three diffusion model parameters showed strong rank-order stability. Group-level drift rates tended to increase, whereas group-level boundary separations decreased and group-level non-decision times exhibited no clear change. These findings could be interpreted as practice effects, which is remarkable given the long time intervals between the sessions (up to one year). People differed from one another in their base rates of all three main diffusion model parameters (intercepts in the growth curve models), but only drift rates showed inter-individual differences in change across time (slopes). Profiles of the three parameters mostly stayed stable across time, but some participants showed strong deviations from this pattern. We believe our study makes a strong case for the—with regard to temporal aspects—trait-like qualities of the three core diffusion model parameters. In the light of our results, the use of diffusion model parameters in individual differences research seems warranted and promising.

## Figures and Tables

**Table 1:** Summary statistics of the individual posterior medians of diffusion model parameters for each time point across all groups.

Parameter	Symbol (Time Point)	*N*	*M*	*SD*	Minimum	Maximum
Drift Rate	ν (T1)	359	2.09	0.42	0.82	3.28
	ν (T2)	334	2.21	0.46	0.94	4.07
	ν (T3)	293	2.21	0.50	0.94	3.82
	ν (T4)	282	2.21	0.50	0.98	3.65
Boundary Separation	*a* (T1)	359	2.04	0.55	1.21	4.79
	*a* (T2)	334	1.89	0.51	1.03	3.98
	*a* (T3)	293	1.87	0.54	0.99	4.04
	*a* (T4)	282	1.85	0.56	0.97	4.39
Non-Decision Time	τ (T1)	359	0.43	0.08	0.29	0.72
	τ (T2)	334	0.42	0.08	0.28	0.78
	τ (T3)	293	0.44	0.09	0.25	0.72
	τ (T4)	282	0.43	0.09	0.27	0.75

Note: *M* = mean. *SD* = standard deviation. Time 2 = Time 1 + 6 months. Time 3 = Time 1 + 12 months. Time 4 = Time 1 + 24 months.

**Table 2:** Correlation matrices of diffusion model parameters across four time points across all participants.

	Time 1	Time 2	Time 3
ν Time 2	0.79 [0.76–0.82]
ν Time 3	0.73 [0.69–0.78]	0.78 [0.75–0.82]
ν Time 4	0.64 [0.59–0.70]	0.71 [0.66–0.76]	0.71 [0.65–0.76]
*a* Time 2	0.85 [0.82–0.87]
*a* Time 3	0.83 [0.80–0.86]	0.90 [0.88–0.91]
*a* Time 4	0.84 [0.82–0.87]	0.88 [0.86–0.91]	0.85 [0.82–0.88]
τ Time 2	0.88 [0.86–0.90]
τ Time 3	0.87 [0.84–0.89]	0.90 [0.88–0.92]
τ Time 4	0.80 [0.76–0.83]	0.86 [0.83–0.88]	0.84 [0.81–0.87]

Note: Means of Bayesian correlation estimates and 95% credible interval are reported. All Bayes factors are >999. Time 2 = Time 1 + 6 months. Time 3 = Time 1 + 12 months. Time 4 = Time 1 + 24 months.

**Table 3:** Parameter estimates and model fit of the drift rate growth curve model.

	Variable	Estimate	Posterior SD	95% CI	Std. Est.
Loadings Intercept	ν (T1)	1.000	-	0.944
	ν (T2)	1.000	-	0.852
	ν (T3)	1.000	-	0.797
	ν (T4)	1.000	-	0.751
Loadings Slope	ν (T1)	0.000	-	0.000
	ν (T2)	1.000	-	0.340
	ν (T3)	1.142	0.143	0.875–1.439	0.364
	ν (T4)	1.297	0.177	0.974–1.668	0.389
(Residual) Variances	ν (T1)	0.020	0.007	0.008–0.033	0.110
	ν (T2)	0.036	0.005	0.026–0.046	0.158
	ν (T3)	0.060	0.007	0.046–0.075	0.233
	ν (T4)	0.082	0.010	0.064–0.103	0.284
	I	0.164	0.014	0.139–0.191	1.000
	S	0.026	0.007	0.014–0.041	1.000
Covariance I and S		0.000	-	0.000
Intercepts	ν (T1)	0.000	-	0.000
	ν (T2)	0.000	-	0.000
	ν (T3)	0.000	-	0.000
	ν (T4)	0.000	-	0.000
	I	2.104	0.022	2.06–2.148	5.202
	S	0.112	0.015	0.081–0.142	0.691
bCFI = 0.998, bGammaHat = 0.997					

Bayesian parameter estimates. Std. Est = completely standardized solution. I = latent intercept. S = latent slope. CI = credible interval.

**Table 4:** Parameter estimates and model fit of the boundary separation growth curve model.

	Variable	Estimate	Posterior SD	95% CI	Std. Est.
Loadings Intercept	*a* (T1)	1.000	-	0.906
	*a* (T2)	1.000	-	0.966
	*a* (T3)	1.000	-	0.939
	*a* (T4)	1.000	-	0.927
Loadings Slope	*a* (T1)	0.000	-	0.000
	*a* (T2)	1.000	-	0.000
	*a* (T3)	1.233	0.127	1.008–1.505	0.000
	*a* (T4)	1.334	0.142	1.077–1.638	0.000
(Residual) Variances	*a* (T1)	0.060	0.006	0.049–0.072	0.180
	*a* (T2)	0.020	0.003	0.014–0.026	0.067
	*a* (T3)	0.036	0.004	0.029–0.046	0.118
	*a* (T4)	0.045	0.005	0.036–0.055	0.141
	I	0.274	0.021	0.235–0.318	1.000
	S	0.000	-	0.000
Covariance I and S		0.000	-	0.000
Intercepts	*a* (T1)	0.000	-	0.000
	*a* (T2)	0.000	-	0.000
	*a* (T3)	0.000	-	0.000
	*a* (T4)	0.000	-	0.000
	I	2.053	0.030	1.995–2.111	3.922
	S	−0.123	0.015	−0.153–−0.093	-Inf
bCFI = 0.999, bGammaHat = 0.999					

Bayesian parameter estimates. Std. Est = completely standardized solution. I = latent intercept. S = latent slope. CI = credible interval.

**Table 5:** Parameter estimates and model fit of the non-decision time growth curve model.

	Variable	Estimate	Posterior SD	95% CI	Std. Est.
Loadings Intercept	τ (T1)	1.000	-	0.932
	τ (T2)	1.000	-	0.967
	τ (T3)	1.000	-	0.931
	τ (T4)	1.000	-	0.894
Loadings Slope	τ (T1)	0.000	-	0.000
	τ (T2)	1.000	-	0.000
	τ (T3)	−0.358	0.354	−1.216–0.157	−0.000
	τ (T4)	0.509	0.291	−0.092–1.053	0.000
(Residual) Variances	τ (T1)	0.001	0.000	0.001–0.001	0.131
	τ (T2)	0.000	0.000	0–0.001	0.066
	τ (T3)	0.001	0.000	0.001–0.001	0.133
	τ (T4)	0.002	0.000	0.001–0.002	0.201
	I	0.006	0.000	0.005–0.007	1.000
	S	0.000	-	0.000
Covariance I and S		0.000	-	0.000
Intercepts	τ (T1)	0.000	-	0.000
	τ (T2)	0.000	-	0.000
	τ (T3)	0.000	-	0.000
	τ (T4)	0.000	-	0.000
	I	0.436	0.004	0.428–0.445	5.526
	S	−0.010	0.002	−0.014–−0.006	-Inf
bCFI = 0.984, bGammaHat = 0.971					

Note: Bayesian parameter estimates. Std. Est = completely standardized solution. I = latent intercept. S = latent slope. CI = credible interval.

**Table 6:** Descriptives of *q* correlations of main diffusion model parameters across time.

Time	Mean	*SD*	Median	*N*
Time 1–Time 2	0.73	0.43	0.91	318
Time 1–Time 3	0.68	0.46	0.89	286
Time 1–Time 4	0.65	0.47	0.86	275
Time 2–Time 3	0.70	0.50	0.93	277
Time 2–Time 4	0.68	0.48	0.91	268
Time 3–Time 4	0.66	0.53	0.91	249

**Figure A1:**
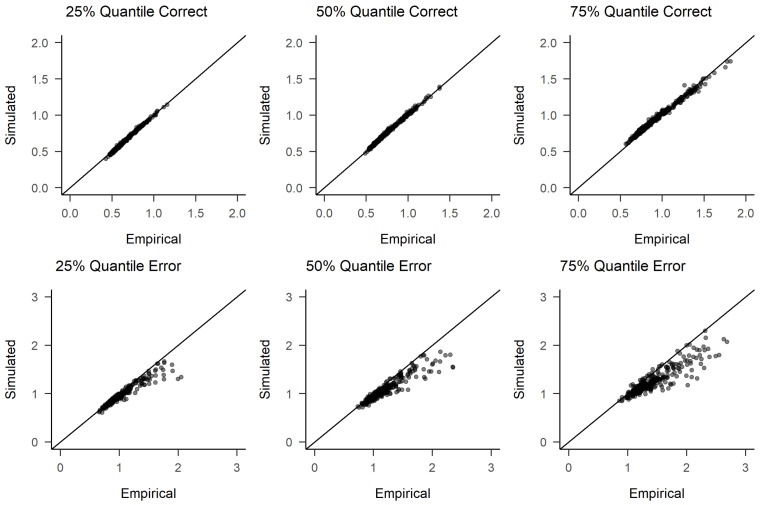
Posterior predictive check of RTs for T2. Participants with 10 or less errors are omitted from the error response time plots.

**Figure A2:**
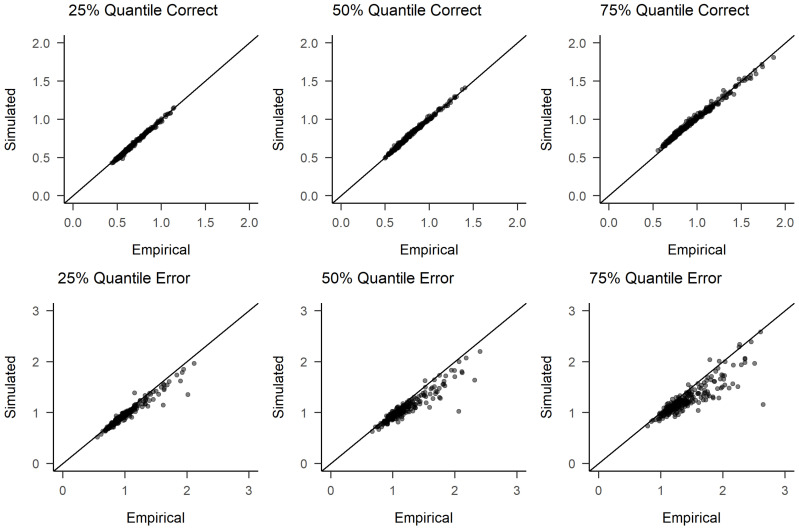
Posterior predictive check of RTs for T3. Participants with 10 or less errors are omitted from the error response time plots.

**Figure A3:**
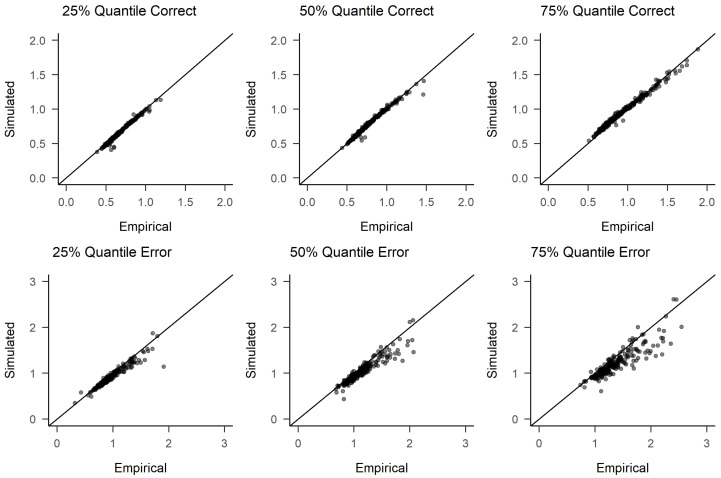
Posterior predictive check of RTs for T4. Participants with 10 or less errors are omitted from the error response time plots.

**Figure A4:**
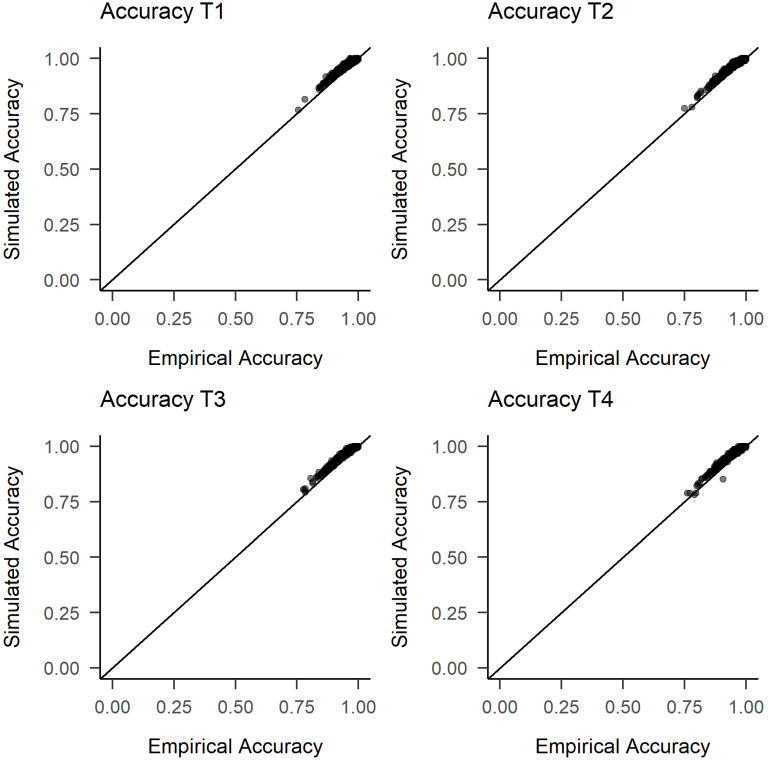
Posterior predictive checks of accuracy rates for all time points.

## Data Availability

Data are available at https://osf.io/cnr2a, accessed on 11 May 2021.

## Appendix A

**Table A1:** Prior distributions used in all analyses.

Parameter	Prior
Diffusion model parameters
$\mu_{a}$	Gamma(1.5,0.75)
$\sigma_{a}$	Half−normal(0.1)
$a_{j}$	$Gamma(\mu_{a},\sigma_{a}^{2})$
$\mu_{\nu }$	Normal(2,3)
$\sigma_{\nu }$	Half−normal(2)
$v_{j}$	$Normal(\mu_{\nu },\sigma_{\nu }^{2})$
$\mu_{\tau }$	Gamma(0.4,0.2)
$\sigma_{\tau }$	Half−normal(1)
$\tau_{j}$	$Normal(\mu_{\tau },\sigma_{\tau }^{2})$
Growth curve model
Factor loading	Normal(0,10)
Latent variable covariance	LKJcorrelation(1)
Latent Intercept	Normal(0,10)
Latent SD	Gamma(1,0.5)
All correlations	Beta(1,1)

Note: The diffusion model parameters are HDDM standards based on the suggestions by Matzke and Wagenmakers (2009). The index *j* refers to individual participants (at a certain time point).

**Table A2:** Summary statistics of the individual diffusion model parameter estimates for each time point for Group 1.

Parameter	Symbol (Time Point)	*n*	*M*	*SD*	Minimum	Maximum
Drift Rate	ν (T1)	112	2.12	0.45	1.14	3.28
	ν (T2)	102	2.21	0.46	1.24	3.38
	ν (T3)	93	2.16	0.49	1.31	3.63
	ν (T4)	89	2.16	0.47	1.05	3.46
Boundary Separation	*a* (T1)	112	1.76	0.32	1.23	2.91
	*a* (T2)	102	1.64	0.28	1.03	2.48
	*a* (T3)	93	1.58	0.26	0.99	2.22
	*a* (T4)	89	1.54	0.26	0.97	2.26
Non-Decision Time	τ (T1)	112	0.39	0.04	0.29	0.48
	τ (T2)	102	0.38	0.03	0.30	0.46
	τ (T3)	93	0.39	0.04	0.29	0.49
	τ (T4)	89	0.38	0.04	0.28	0.49

Note: *M* = mean. *SD* = standard deviation. Individual posterior medians used.

**Table A3:** Summary statistics of the individual diffusion model parameter estimates for each time point for Group 2.

Parameter	Symbol (Time Point)	*n*	*M*	*SD*	Minimum	Maximum
Drift Rate	ν (T1)	103	2.01	0.39	1.08	2.88
	ν (T2)	104	2.15	0.45	1.24	3.29
	ν (T3)	85	2.08	0.42	1.12	3.14
	ν (T4)	82	2.11	0.46	1.20	3.36
Boundary Separation	*a* (T1)	103	1.78	0.34	1.21	3.60
	*a* (T2)	104	1.65	0.35	1.08	3.40
	*a* (T3)	85	1.60	0.27	1.15	2.20
	*a* (T4)	82	1.58	0.29	1.03	2.24
Non-Decision Time	τ (T1)	103	0.40	0.05	0.30	0.51
	τ (T2)	104	0.39	0.05	0.28	0.52
	τ (T3)	85	0.39	0.05	0.25	0.51
	τ (T4)	82	0.38	0.04	0.27	0.55

Note: *M* = mean. *SD* = standard deviation. Individual posterior medians used.

**Table A4:** Summary statistics of the individual diffusion model parameter estimates for each time point for Group 3.

Parameter	Symbol (Time Point)	*n*	*M*	*SD*	Minimum	Maximum
Drift Rate	ν (T1)	26	1.93	0.43	1.28	3.15
	ν (T2)	23	2.02	0.49	1.30	3.13
	ν (T3)	18	1.93	0.58	0.99	3.50
	ν (T4)	20	1.87	0.44	1.08	2.68
Boundary Separation	*a* (T1)	26	1.88	0.36	1.38	2.98
	*a* (T2)	23	1.72	0.27	1.23	2.24
	*a* (T3)	18	1.74	0.27	1.22	2.14
	*a* (T4)	20	1.72	0.36	1.27	2.62
Non-Decision Time	τ (T1)	26	0.40	0.06	0.30	0.52
	τ (T2)	23	0.39	0.05	0.30	0.48
	τ (T3)	18	0.38	0.05	0.31	0.47
	τ (T4)	20	0.40	0.06	0.30	0.53

Note: *M* = mean. *SD* = standard deviation. Individual posterior medians used.

**Table A5:** Summary statistics of the individual diffusion model parameter estimates for each time point for Group 4.

Parameter	Symbol (Time Point)	*n*	*M*	*SD*	Minimum	Maximum
Drift Rate	ν (T1)	58	2.23	0.45	0.82	3.24
	ν (T2)	55	2.30	0.47	0.94	4.07
	ν (T3)	44	2.45	0.57	0.94	3.82
	ν (T4)	44	2.39	0.52	0.98	3.65
Boundary Separation	*a* (T1)	58	2.59	0.64	1.79	4.79
	*a* (T2)	55	2.44	0.51	1.61	3.88
	*a* (T3)	44	2.42	0.54	1.56	4.04
	*a* (T4)	44	2.44	0.57	1.69	4.39
Non-Decision Time	τ (T1)	58	0.52	0.07	0.36	0.72
	τ (T2)	55	0.52	0.08	0.33	0.78
	τ (T3)	44	0.55	0.08	0.36	0.72
	τ (T4)	44	0.53	0.07	0.37	0.71

Note: *M* = mean. *SD* = standard deviation. Individual posterior medians used.

**Table A6:** Summary statistics of the individual diffusion model parameter estimates for each time point for Group 5.

Parameter	Symbol (Time Point)	*n*	*M*	*SD*	Minimum	Maximum
Drift Rate	ν (T1)	53	2.12	0.38	1.25	2.90
	ν (T2)	48	2.36	0.41	1.73	3.65
	ν (T3)	51	2.42	0.39	1.77	3.27
	ν (T4)	44	2.45	0.47	1.12	3.36
Boundary Separation	*a* (T1)	53	2.56	0.44	1.75	3.56
	*a* (T2)	48	2.40	0.41	1.74	3.98
	*a* (T3)	51	2.44	0.52	1.66	3.93
	*a* (T4)	44	2.46	0.50	1.71	4.07
Non-Decision Time	τ (T1)	53	0.51	0.07	0.38	0.66
	τ (T2)	48	0.50	0.07	0.36	0.62
	τ (T3)	51	0.52	0.07	0.37	0.64
	τ (T4)	44	0.52	0.09	0.28	0.75

Note: *M* = mean. *SD* = standard deviation. Individual posterior medians used.

**Table A7:** Correlation matrix of drift rates across four time points split by groups.

Time Point	Group	Time 1	Time 2	Time 3
ν Time 2	Group 1	0.77 [0.70–0.83]		
ν Time 3		0.70 [0.61–0.78]	0.71 [0.63–0.79]	
ν Time 4		0.63 [0.53–0.73]	0.66 [0.57–0.76]	0.62 [0.51–0.73]
ν Time 2	Group 2	0.80 [0.74–0.85]		
ν Time 3		0.69 [0.60–0.79]	0.79 [0.72–0.85]	
ν Time 4		0.66 [0.57–0.77]	0.76 [0.68–0.83]	0.66 [0.55–0.76]
ν Time 2	Group 3	0.72 [0.55–0.87]		
ν Time 3		0.76 [0.59–0.91]	0.86 [0.76–0.95]	
ν Time 4		0.47 [0.20–0.74]	0.46 [0.17–0.72]	0.82 [0.68–0.95]
ν Time 2	Group 4	0.80 [0.72–0.88]		
ν Time 3		0.77 [0.68–0.87]	0.86 [0.79–0.92]	
ν Time 4		0.70 [0.57–0.81]	0.85 [0.76–0.91]	0.76 [0.64–0.87]
ν Time 2	Group 5	0.80 [0.70–0.88]		
ν Time 3		0.77 [0.67–0.86]	0.80 [0.70–0.87]	
ν Time 4		0.55 [0.38–0.73]	0.55 [0.37–0.73]	0.57 [0.40–0.74]

Note: Means of Bayesian correlation estimates and 95% credible interval are reported.

**Table A8:** Correlation matrix of boundary separation across four time points split by groups.

Time Point	Group	Time 1	Time 2	Time 3
*a* Time 2	Group 1	0.69 [0.60–0.77]		
*a* Time 3		0.72 [0.64–0.80]	0.81 [0.75–0.86]	
*a* Time 4		0.65 [0.55–0.75]	0.71 [0.63–0.80]	0.77 [0.69–0.83]
*a* Time 2	Group 2	0.85 [0.80–0.89]		
*a* Time 3		0.67 [0.58–0.77]	0.84 [0.79–0.89]	
*a* Time 4		0.69 [0.59–0.78]	0.77 [0.70–0.84]	0.83 [0.77–0.89]
*a* Time 2	Group 3	0.70 [0.51–0.86]		
*a* Time 3		0.69 [0.46–0.87]	0.87 [0.77–0.96]	
*a* Time 4		0.79 [0.63–0.91]	0.90 [0.81–0.96]	0.79 [0.59–0.92]
*a* Time 2	Group 4	0.69 [0.57–0.81]		
*a* Time 3		0.61 [0.44–0.76]	0.76 [0.66–0.87]	
*a* Time 4		0.73 [0.61–0.84]	0.81 [0.71–0.88]	0.60 [0.43–0.78]
*a* Time 2	Group 5	0.60 [0.44–0.74]		
*a* Time 3		0.63 [0.49–0.77]	0.72 [0.60–0.84]	
*a* Time 4		0.58 [0.40–0.74]	0.62 [0.45–0.77]	0.58 [0.39–0.72]

Note: Means of Bayesian correlation estimates and 95% credible interval are reported.

**Table A9:** Correlation matrix of non-decision times across four time points split by groups.

Time Point	Group	Time 1	Time 2	Time 3
τ Time 2	Group 1	0.68 [0.60–0.76]		
τ Time 3		0.63 [0.53–0.73]	0.61 [0.50–0.71]	
τ Time 4		0.55 [0.43–0.66]	0.57 [0.45–0.68]	0.62 [0.50–0.72]
τ Time 2	Group 2	0.72 [0.64–0.80]		
τ Time 3		0.59 [0.47–0.70]	0.77 [0.70–0.84]	
τ Time 4		0.49 [0.35–0.62]	0.65 [0.54–0.74]	0.66 [0.56–0.76]
τ Time 2	Group 3	0.78 [0.65–0.91]		
τ Time 3		0.73 [0.53–0.89]	0.64 [0.41–0.84]	
τ Time 4		0.68 [0.47–0.86]	0.73 [0.54–0.88]	0.60 [0.31–0.82]
τ Time 2	Group 4	0.71 [0.60–0.82]		
τ Time 3		0.68 [0.54–0.81]	0.75 [0.62–0.84]	
τ Time 4		0.50 [0.33–0.69]	0.70 [0.56–0.82]	0.51 [0.31–0.71]
τ Time 2	Group 5	0.71 [0.58–0.82]		
τ Time 3		0.73 [0.62–0.84]	0.83 [0.75–0.90]	
τ Time 4		0.58 [0.43–0.75]	0.70 [0.56–0.83]	0.67 [0.51–0.79]

Note: Means of Bayesian correlation estimates and 95% credible interval are reported.

**Table A10:** Correlation matrix of raw data across four time points across all groups.

Time Point	Time 1	Time 2	Time 3
Accuracy Time 2	0.85 [0.82–0.87]
Accuracy Time 3	0.82 [0.79–0.85]	0.88 [0.86–0.90]
Accuracy Time 4	0.77 [0.73–0.80]	0.84 [0.82–0.87]	0.87 [0.85–0.89]
Median correct RT Time 2	0.95 [0.94–0.96]
Median correct RT Time 3	0.92 [0.91–0.94]	0.96 [0.95–0.97]
Median correct RT Time 4	0.89 [0.87–0.91]	0.91 [0.89–0.92]	0.92 [0.90–0.93]
Median error RT Time 2	0.85 [0.83–0.87]
Median error RT Time 3	0.82 [0.79–0.85]	0.87 [0.85–0.89]
Median error RT Time 4	0.83 [0.80–0.85]	0.85 [0.82–0.87]	0.84 [0.81–0.86]

Note: Means of Bayesian correlation estimates and 95% credible interval are reported.
